# Comparative histomorphological assessment of the osteoinductive capacity of a nanofibrillated cellulose‐based composite and autologous blood clot

**DOI:** 10.1002/jeo2.70067

**Published:** 2024-11-05

**Authors:** Berik Tuleubayev, Yevgeniy Kamyshanskiy, Dina Saginova, Saule Akhmetova, Vladimir Vinokurov, Amina Koshanova, Yekaterina Kossilova

**Affiliations:** ^1^ Department of Surgical Diseases Karaganda Medical University Karaganda Kazakhstan; ^2^ Pathology Unit of the University Clinic Karaganda Medical University Karaganda Kazakhstan; ^3^ Center for Applied Scientific Research National Scientific Center of Traumatology and Orthopaedics Named after Academician N.D. Batpenov Astana Kazakhstan; ^4^ Department of Clinical Immunology Karaganda Medical University Karaganda Kazakhstan; ^5^ Department of Physical and Colloidal Chemistry Federal State Autonomous Educational Institution of Higher Education Gubkin Russian State University of Oil and Gas, National Research University Moscow Russian Federation

**Keywords:** autologous blood clot, bone defect, bone regeneration, composite, nanocellulose

## Abstract

**Purpose:**

The present study aimed to evaluate and compare the effect of nanofibrillated cellulose (NFC)‐based composite with dicalcium phosphate dihydrate and an autologous blood clot (ABC) on the formation of new bone tissue in vivo by histological and histomorphometric assessment.

**Materials and Methods:**

A total of 72 rats with created femoral defects (2 mm) were used. The rats were divided into three groups: (1) with filling of the defect with an ABC, (2) NFC‐1—with filling of both the cortical plate and intramedullary space in the defect area, and (3) NFC‐2—with filling of only the intramedullary space in the defect area. Histological and histomorphometric analysis was performed to assess the healing of the bone defect after 14, 30 and 60 days.

**Results:**

Complete closure of the cortical plate defect was detected in the NFC‐2 group on Day 30 (*p* < 0.0001). Moreover, in both NFC groups on the 30th and 60th days, ongoing osteogenesis was observed, characterized by a large volume of newly formed circular pattern bone tissue in the intramedullary space.

**Сonclusion:**

This study demonstrated that the NFC‐based composite, which is located below the level of the cortical plate, tamponing only the intramedullary space (NFC‐2), improves bone tissue repair at the site of a bone defect of the cortical plate and has the potential of prolonged osteoinductivity.

**Level of Evidence:**

Not applicable.

**Abbreviations**
ABC: autologous blood clot
H&E: hematoxylin and eosin
NFC: nanofibrillated cellulose‐based composite with dicalcium phosphate dihydrate
NFC‐1: with filling of both the cortical plate and intramedullary space in the defect area
NFC‐2: with filling of only the intramedullary space in the defect area

## INTRODUCTION

It is well known that small bone defects can regenerate on their own, while extensive bone injuries, in which a bone defect is not capable of self‐regeneration, require surgical reconstruction [2, 26, 49, 50]. In recent years, the number of methods for treating bone diseases and defects has increased, as well as the growing need for the use of various materials for bone regeneration [15, 20, 29, 34, 43].

Autologous bone graft is the preferred material for filling bone defects, but its disadvantage is the inability to fully fill in the large defects due to its poor postoperative survival rate, as well as the burden of cosmetic defects and additional surgery [12, 33]. Currently, in modern traumatology and orthopaedics, when choosing a bone defect filling method and material, more and more attention is paid to nanomaterials and composites based on them. The main requirement for bone regeneration materials is the simultaneous achievement of biological activity, biocompatibility, mechanical properties and ease of manufacture [42]. Nanofiber cellulose (NFC) are crystalline nanoparticles isolated from natural cellulose through an acid hydrolysis procedure [13]. Nanocellulose is used to produce nanocomposites with excellent mechanical properties for various biomedical applications [41]. These composites combine the key properties of cellulose, such as high specific strength and elasticity, chemical inertia and hydrophilicity [6, 19]. In addition, nanocellulose has excellent barrier and antimicrobial properties [3, 10], high biodegradability and availability due to renewable resources, and low manufacturing and processing costs [16]. However, the limiting factor for the use of nanocellulose as a bone graft is the lack of biological activity that causes bone regeneration.

The development of composite biomaterials to meet the needs of bone tissue engineering has become an important area of recent research. Several studies have reported improvements in the properties of bone scaffolds based on nanoscale additives, such as nanoscale calcium silicate [44] or carbon nanotubes [30]. The observed improvements include better mechanical properties and biomineralization [17, 46]. Composite biomaterials are primarily designed to enhance the degradation rate, mechanical properties and biological activity of bone scaffolds [4, 39]. Accordingly, nanocellulose‐based composites have a high chance of becoming ideal candidates for use in regenerative medicine and bone engineering.

The purpose of this study was to conduct a comparative histological and histomorphometric assessment of new bone formation using NFC composites and autologous blood clots (ABCs) transplanted into the femoral bone tissue defect in an animal experimental model.

## MATERIALS AND METHODS

### Preparation of nanofibrillated cellulose‐based composite with dicalcium phosphate dihydrate

NFC is the result of multistage chemical and mechanical processing of nanocellulose, which results in the formation of nanofibers with diameters ranging from 5 to 100 nm and lengths ranging from 200 nm to several tens of microns. The specific surface area of such nanofibers is 100–200 m^2^/g. NFC is a nanoscale cellulose fibre that contains amorphous and crystalline regions with a high length‐to‐diameter ratio [1]. In this study, a nanocellulose‐based biocomposite was used, which was prepared from nanocellulose with dicalcium phosphate dihydrate (CaHPO_4_·2H_2_O) was obtained as follows: bleached cellulose, which is a mass in the form of white fibres, which were subsequently subjected to mechanical destruction at the Masuko MKCA supermass 6‐5 collider for eight cycles. Then, the final product was NFC with an average fibre diameter (fibrils) of 12 nm and an average length of 1.5 µm, after which the synthesis of a composite (NFC) and dicalcium phosphate dihydrate began. Two solutions were prepared for this purpose. The first solution was prepared by dissolving 0.05 mol of Na_2_HPO_4_·2H_2_O in 300 mL of an aqueous suspension of NFC in water, and the second solution was prepared by dissolving 0.05 mol of Ca(NO_3_·6H_2_O in 100 mL of distilled water. The second solution was added dropwise to a solution of sodium phosphate with a suspension of NFC, maintaining a pH from 6 to 6.5 using a 25% ammonia solution. After that, the resulting solutions were stirred (at 400 rpm) at room temperature for 1 h. The resulting precipitate was filtered under vacuum on filter paper through a Buchner funnel, washed three times with deionized water and dried overnight at 40°C in a drying cabinet.

### Animals and surgical procedures

**Figure 1:**
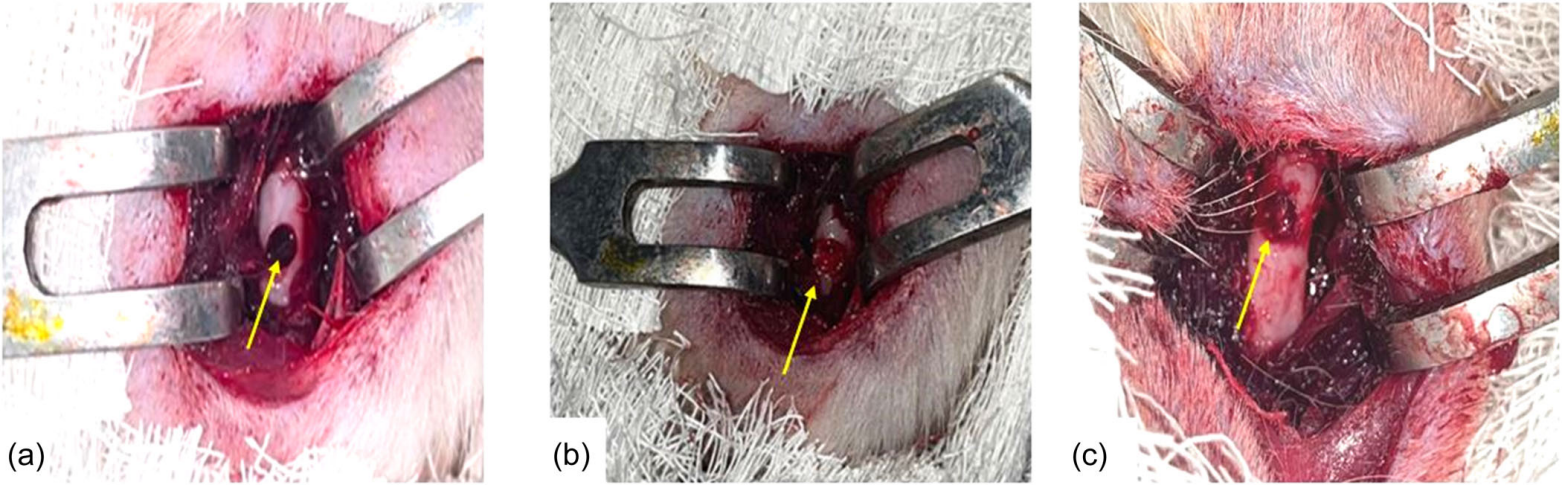
Macroscopic intraoperative view of the femoral bone defect model in groups with defect filling with autologous blood clot (ABC) (a) and nanocellulose with different filling techniques: (b) NFC‐1—with filling of both the cortical plate and intramedullary space in the defect area, (c) NFC‐2—with filling of only the intramedullary space in the defect area. (a) Macroscopic view of filling a femoral bone defect with an ABC—the defect is well visualized, the edges of the defect are even and the defect cavity is filled with a dark‐red mass (ABC) (yellow arrow). (b) Macroscopic view of a cortical plate and intramedullary space in the defect area filled with nanocellulose using the NFC‐1 filling technique ‐ the edges of the defect are not visualized, the synthetic nanocellulose‐based composite with dicalcium phosphate dihydrate protrudes from the defect above the level of the cortical plate, completely obturates the intramedullary space and the cortical plate in the defect area (yellow arrow). (c) Macroscopic view of the cortical plate and intramedullary space in the defect area filled with nanocellulose using the NFC‐2 filling technique—the edges of the defect are well visualized and even. Nanocellulose‐based composite with dicalcium phosphate dihydrate is located below the level of the cortical plate, filling only the intramedullary space (yellow arrow).

The animals are randomly divided into three equal groups of 24 rats each. Under anaesthesia with zoletil at a dose of 0.1 mg/kg (Virbac), an operative incision was made on the anterior surface of the thigh with a length of about 10 mm, the femur was exposed, and a rounded bone defect with a diameter of 2 mm and a depth of 1 mm was formed in the projection of the thigh. the middle third of the diaphysis of the femur using a drill (Figure 1a) [22, 38]. In the first group (positive control), the defect site was filled with ABCs. To do this, the blood obtained during the incision or after the formation of a bone defect in a 1 mL polypropylene syringe without any anticoagulant was taken from the rats of the control group (without the use of nanocellulose) during the surgical procedure, after which thrombin was added to the blood sample to stimulate the formation of a clot and the formed clot was applied to the bone defect.

In the second and third groups, the defect was filled with NFC composite in two different ways. In the second group (NFC‐1)—with filling of both the cortical plate and intramedullary space in the defect area (Figure 1b). In the third group (NFC‐2 with filling of only the intramedullary space in the defect area (Figure 1c). After surgery, the wounds were sutured with Vicryl 5.0 (Ethicon, Johnson & Johnson). After surgery, each animal received intramuscular Ketonal injections of 0.04 mL/kg (Sandoz) for 3 days. The clinical condition of the rats was assessed by measuring the temperature, weight and general motor activity of the animals, as well as the degree of wound healing. Wound healing was considered satisfactory in the absence of signs of inflammation, marginal necrosis and complete preservation of sutures. On the 14th, 30th and 60th days after the operation, the experimental animals were euthanized from an overdose of knocked up. After that, a dissection of the operated limb was performed to collect material (a graft with bone and surrounding muscles) for histological examination.

### Histopathological examination

Before the histological analysis samples were fixed in 10% neutral buffered formalin for 24 h, followed by decalcification in a solution of Biodec R (Bio‐Optica Milano SPA) for another 24 h. Then the samples were rinsed in phosphate buffer (pH = 7.4). After achieving optimal softening of the bone tissue (decalcification), a bone section and orientation in the histological block were made. The tissue was fixed in 10% formalin at 4°C for 24 h, rinsed with tap water and dehydrated using a series of alcohols of increasing concentrations (70%, 90%, 95% and 100%). The samples were then immersed in xylene and embedded in paraffin blocks. Tissue sections with a thickness of 5 µm were prepared using a Leica SM 2000R sliding microtome. After preparation, tissue sections were stained with hematoxylin and eosin (H&E) for general morphological analysis of the tissue, detection of inflammatory infiltration and necrosis, and with Masson's trichrome to assess bone graft remodelling and new bone formation [5].

### H&E staining procedure

The tissue sections were immersed in Mayer's hematoxylin for a quarter of an hour and then rinsed with tap water for 5 min. After that, the sections were stained with eosin for a minute.

### Masson's trichrome staining procedure

A commercial kit was used for Masson's trichrome staining (Masson's Trichrome BioVitrum TU 9398‐001‐89079081‐2012). After dewaxing and rehydration, the slides were placed in Bouin's solution at a temperature of 56°C for 15 min. This was followed by a 5‐min rinse in tap water. The application of Weigert's hematoxylin lasted 5 min, followed by another 5‐min rinsing in tap water and a quick rinse in distilled water. Then, the slides were stained with Biebrich scarlet‐acid fuchsin solution for 5 mins, rinsed in distilled water and immersed in phosphotungstic‐phosphomolybdic acid for another 5 min. The next step was to apply aniline blue for 5 min, and finally, the slides were fixed in 1% acetic acid for 2 min.

Microscopic examination of slides was performed on a Zeiss AxioLab 4.0 microscope at 400× magnification. The AxioVision 7.2 software for Windows was used to obtain and analyse photomicrographs of the sections.

H&E staining was used to visualize general cell morphology and tissue structure. Masson's trichrome staining was used to identify newly formed bone tissue at different stages of bone defect reparation and to assess bone remodelling.

Two independent researchers with experience in working with animal models conducted a morphometric study without knowing which group each animal belonged to. The terminology used in histomorphometric analysis corresponded to the recommendations of the Histomorphometry Nomenclature Committee of the American Society for Bone and Mineral Research [8]. The following parameters were defined: (1) closure of the cortical plate defect with newly formed bone tissue in length and thickness, in %; (2) tissue composition of the cortical plate and intramedullary space in the defect area, in %.

Morphometric assessment of cortical plate tissue was performed within the area radially bounded by the defect edges and laterally by the native femur, as well as by the outer boundary of the bone graft and/or newly formed bone tissue. This estimate was presented as a percentage of the total defect area. Three histological sections were analysed for each bone defect and their average was calculated. Tissues indicating a non‐specific reparative process, such as vessels or the Haversian canals, were not included in the quantitative assessment and accounted for the smallest percentage in the area of the bone callus [7, 31].

Morphometric assessment of the intramedullary space tissue was performed within the area of cortical plate defect bounded radially by the cortical plate defect edges projection and laterally by the native femur cortical plate.

### Statistical analysis

All experimental data are presented as averages and standard deviations. Comparison between the two groups was carried out using the chi‐square criterion with Yates continuity correction and the Mann‒Whitney *U* test. Multiple comparisons were carried out using Pearson's chi‐square test. Statistical analysis of the study results was performed using IBM SPSS Statistics 20.0 and STATISTICA 10. A value of *p* < 0.05 was considered statistically significant.

## RESULTS

### The cortical plate

#### Histological and morphometric analysis of newly formed bone tissue

**Figure 2:**
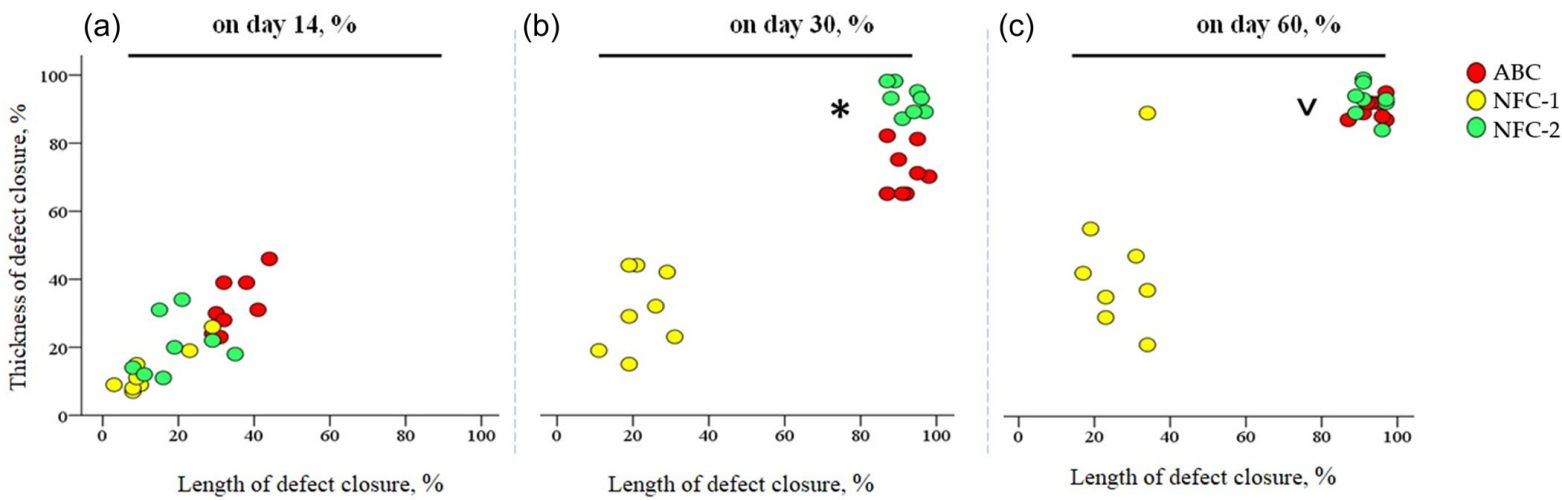
Bone defect closure in a temporal‐spatial manner on 14, 30 and 60 days. NFC‐1—with filling of both the cortical plate and intramedullary space in the defect area, NFC‐2—with filling of only the intramedullary space in the defect area. (a) Partial closure of a cortical plate defect with newly formed bone tissue with regeneration of up to 50% of the defect length and thickness. (b) In the NFC‐2 group, the defect was closed with newly formed bone tissue by more than 80%. In the autologous blood clot (ABC) group, partial regeneration of the defect thickness with complete closure of the length of the cortical plate defect. (c) Complete repair of the cortical plate with full closure of the defect in length and thickness in the NFC‐1 and ABC groups; incomplete and partial regeneration of the cortical plate both in length and thickness in the group utilizing the NFC‐1 defect filling technique. *Statistically significant difference grade of defect closure versus NFC‐1 and ABCs (*p* < 0.05); ˅Statistically significant difference versus NFC‐1 group (*p* < 0.05). NFC, nanofibrillated cellulose‐based composite with dicalcium phosphate dihydrate.

**On Day 14** (Table 1, Figure 2),

**Table 1:** Histopathological assessment of bone defect healing and tissue composition of the intramedullary space in the surgical intervention projection (a) after 14, (b) 30 and (c) 60 days.

	(a)		
	ABC	NFC‐1	NFC‐2
	14 days		
**The cortical plate**			
Closure of the defect with bone tissue, %			
In length	**34.6** ± **5.6**	**12.4** ± **8.8**	**19.3** ± **9.0**
	*р*_1_ = 0.0001	р_3_ = 0.105	
	*р*_2_ = 0.003		
By thickness	**32.5** ± **8.1**	**13.6** ± **6.6**	**20.3** ± **8.5**
	*р*_1_ = 0.001	*р*_3_ = 0.05	
	*р*_2_ = 0.015		
Closure of the defect with other tissues, %			
Autologous blood clot/composite	–	**43.1** ± **6.2**	–
Cartilage tissue	**3.5** ± **2.1**	**5.9** ± **2.5**	**4.6** ± **2.2**
	*р*_1_ = 0.05	*р*_3_ = 0.328	
	*р*_2_ = 0.328		
Fibrous tissue	**6.9** ± **2.8**	**3.5** ± **1.9**	**3.3** ± **1.8**
	*р*_1_ = 0.015	*р*_3_ = 0.878	
	*р*_2_ = 0.007		
**Intramedullary space**			
Autologous blood clot/composite, %	‐	**30.8** ± **9.2**	**20.5** ± **8.5**
		*р*_3_ = 0.04	
Bone tissue, %	**11.4** ± **1.9**	**18.3** ± **4.9**	**29.1** ± **11.3**
	*р*_1_ = 0.015	*р*_3_ = 0.65	
	*р*_2_ = 0.0001		
Cartilage tissue, %	**7.8** ± **4.3**	**9.3** ± **1.2**	**8.4** ± **1.3**
	*р*_1_ = 0.721	*р*_3_ = 0.195	
	*р*_2_ = 0.878		
Fibrous tissue, %	**5.3** ± **1.6**	**9.6** ± **2.1**	**8.1** ± **0.9**
	*р*_1_ = 0.001	*р*_3_ = 0.13	
	*р*_2_ = 0.002		
Bone marrow, %	**75.6** ± **5.4**	**12.3** ± **6.3**	**33.9** ± **16.4**
	*р*_1_ = 0.0001	*р*_3_ = 0.015	
	*р*_2_ = 0.0001		

	**(b)**		
	ABC	NFC‐1	NFC‐2
	**30 days**		
**The cortical plate**			
Closure of the defect with bone tissue, %			
In length	**91.9** ± **3.9**	**21.9** ± **6.5**	**92.1** ± **3.8**
	*р*_1_ = 0.001	*р*_3_ = 0.001	
	*р*_2_ = 0.878		
By thickness	**71.8** ± **6.9**	**31.0** ± **11.5**	**92.8** ± **4.1**
	*р*_1_ = 0.001	р_3_ = 0.001	
	*р*_2_ = 0.001		
Closure of the defect with other tissues, %			
Autologous blood clot/composite	–	**39.9** ± **4.4**	–
Cartilage tissue	**8.1** ± **3.9**	**13.5** ± **5.1**	**3.6** ± **0.9**
	*р*_1_ = 0.065	*р*_3_ = 0.001	
	*р*_2_ = 0.015		
Fibrous tissue	**10.3** ± **3.4**	**9.8** ± **7.9**	**1.6** ± **1.4**
	*р*_1_ = 0.645	*р*_3_ = 0.005	
	*р*_2_ = 0.001		
**Intramedullary space**			
Autologous blood clot/composite, %	–	**29.5** ± **5.1**	**19.0** ± **5.5**
		*р*_3_ = 0.002	
Bone tissue, %	**5.0** ± **1.2**	**29.7** ± **0.7**	**42.3** ± **3.5**
	*р*_1_ = 0.001	*р*_3_ = 0.001	
	*р*_2_ = 0.001		
Cartilage tissue, %	**2.6** ± **2.1**	**19.1** ± **2.1**	**9.1** ± **1.4**
	*р*_1_ = 0.001	*р*_3_ = 0.001	
	*р*_2_ = 0.001		
Fibrous tissue, %	**1.4** ± **2.1**	**17.1** ± **2.1**	**8.3** ± **1.9**
	*р*_1_ = 0.001	*р*_3_ = 0.001	
	*р*_2_ = 0. 001		
Bone marrow, %	**91.0** ± **3.4**	**4.5** ± **2.5**	**21.4** ± **6.9**
	*р*_1_ = 0.001	*р*_3_ = 0.001	
	*р*_2_ = 0.001		

	(c)		
	ABC	NFC‐1	NFC‐2
	60 days		
**The cortical plate**			
Closure of the defect with bone tissue, %			
In length	**93.5** ± **3.4**	**26.9** ± **7.1**	**92.6** ± **3.4**
	*р*_1_ = 0.001	*р*_1_ = 0.001	
	*р*_2_ = 0.574		
By thickness	**90.3** ± **2.9**	**44.4** ± **20.8**	**92.8** ± **4.8**
	*р*_1_ = 0.001	*р*_1_ = 0.001	
	*р*_2_ = 0.161		
Closure of the defect with other tissues, %			
Autologous blood clot/composite	–	**42.3** ± **5.6**	–
Cartilage tissue	**4.0** ± **1.5**	**18.9** ± **9.3**	**3.9** ± **3.4**
	*р*_1_ = 0.003	*р*_3_ = 0.05	
	*р*_2_ = 0.574		
Fibrous tissue	**2.9** ± **0.8**	**20.3** ± **15.5**	**1.9** ± **1.8**
	*р*_1_ = 0.001	*р*_3_ = 0.001	
	*р*_2_ = 0.279		
**Intramedullary space**			
Autologous blood clot/composite, %	–	**32.3** ± **18.1**	**19.1** ± **8.2**
		*р*_3_ = 0.028	
Bone tissue, %	**4.1** ± **1.7**	**32.0** ± **22.7**	**47.6** ± **15.7**
	*р*_1_ = 0.001	*р*_3_ = 0.442	
	*р*_2_ = 0.001		
Cartilage tissue, %	**1.4** ± **2.1**	**13.3** ± **8.7**	**8.4** ± **1.4**
	*р*_1_ = 0.001	*р*_3_ = 0.878	
	*р*_2_ = 0.001		
Fibrous tissue, %	**0.8** ± **0.7**	**14.6** ± **7.4**	**9.8** ± **1.9**
	*р*_1_ = 0.001	*р*_3_ = 0.105	
	*р*_2_ = 0.001		
Bone marrow, %	**93.8** ± **2.8**	**7.9** ± **8.6**	**15.1** ± **18.7**
	*р*_1_ = 0.001	*р*_3_ = 0.021	
	*р*_2_ = 0.0001		

*Note*: *p* is the significance level; *p*1 < 0.05—statistically significant difference between ABC group and NFC‐1 group, *p*2 < 0.05—statistically significant difference between ABC group and NFC‐2 group and *p*3 < 0.05—statistically significant difference between NFC‐1 group and NFC‐2 group within the intramedullary space.

In the ABC group, the bone defect area was characterized by the development of separate heterogeneous bundles of new bone tissue. The resulting bone trabeculae were uneven, and mostly thin, with focal areas of bone bridges and sporadic contacts, mainly at the ends of the bone trabeculae. The newly formed bone tissue accounted for 34.6% and 32.5% of the length and thickness of the cortical plate, respectively. Other tissues in the defect area accounted for approximately 10%, of which 3.5% were cartilaginous tissue and 6.9% were fibrous tissue.

In the NFC‐1 group, where the composite was at the level of the cortical plate, the newly formed bone tissue in the defect area occupied 12.4% of the length and 13.6% of the thickness of the cortical plate; the composite accounted for 43.1% of the cortical plate defect, cartilaginous tissue amounted to 5.9% and fibrous tissue composed 3.5% of the cortical plate defect.

In the NFC‐2 group, the closure of the defect with newly formed bone tissue was 19.3% of the length and 20.3% of the thickness of the cortical plate; other tissues represented 4.6% of the cartilaginous tissue and 3.3% of the fibrous tissue.

**Figure 3:**
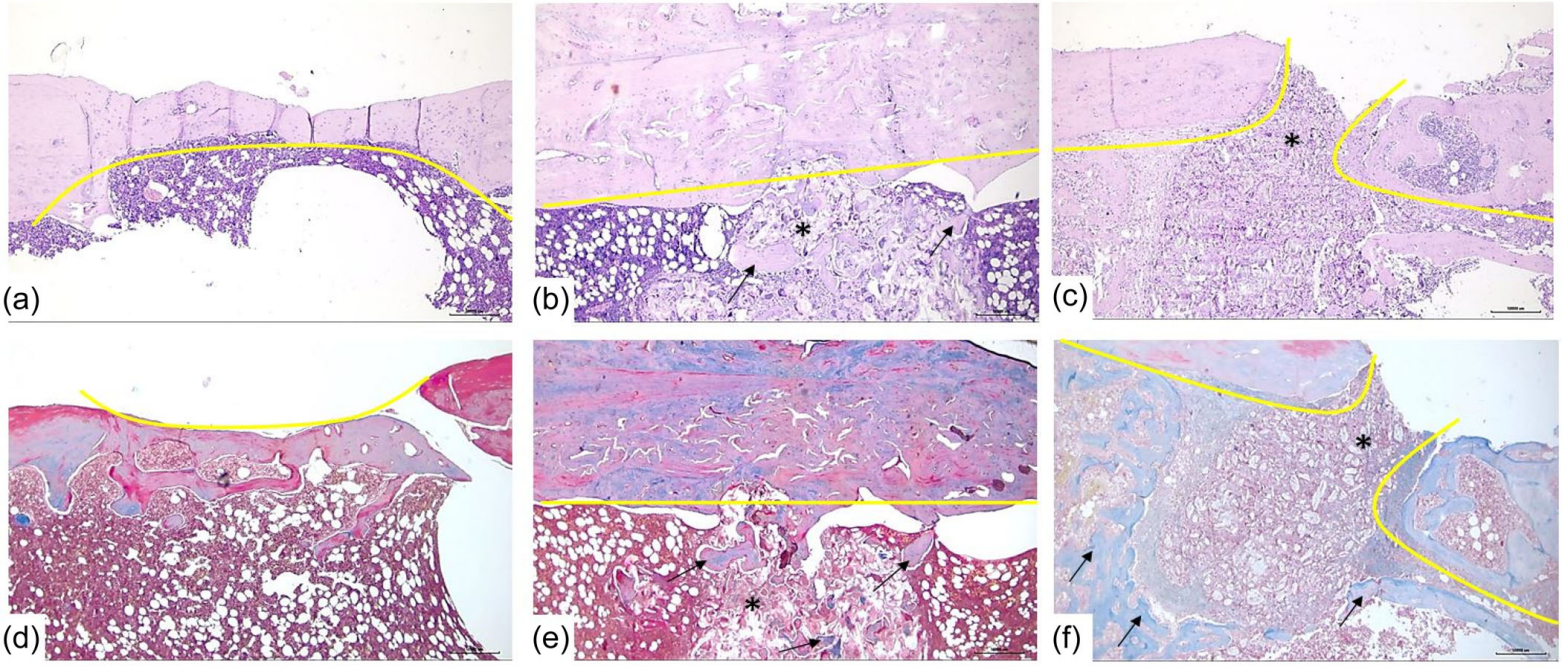
Representative photomicrographs of the defect area of the cortical plate and intramedullary space on the 30th day after filling the defect with an autologous blood clot (ABC) and nanocellulose with different filling techniques. (a, d) Photomicrographs of the defect area with ABC filling—the defect area is partially closed by newly formed bone tissue, with complete regeneration of length continuity and partial regeneration of up to 50% of normal cortical plate thickness in the defect area (in yellow is a curve in the form of a parabola showing the uneven regeneration of the cortical plate thickness). Mature bone marrow in the intramedullary space reflects positive remodelling of the intramedullary space in the defect area with the regeneration of the normal anatomical structure. (b, c, e, f) Photomicrographs of the defect area filled with nanocellulose in various modifications: NFC‐1 and NFC‐2. (b, e) NFC‐2 group: Filling of the intramedullary space defect with nanocellulose without filling the cortical plate defect; relatively uniform regeneration of up to 80% of the cortical plate defect area in length and thickness with newly formed bone tissue (a straight yellow line shows uniform and complete integration of the defect edges with the newly formed bone tissue); in the intramedullary space, under the cortical plate, a nanocellulose‐based composite (asterisk) with a large number of bone trabeculae (arrows) along the periphery and in the centre of the composite is observed, which reflects active osteogenesis. (с, f) NFC‐1 group: Filling the defect with nanocellulose both in the intramedullary space and in the cortical plate; relatively uneven regeneration of up to 40% of the cortical plate defect area in length and thickness with newly formed bone tissue (a discontinuous yellow line shows incomplete integration of the defect edges with the newly formed bone tissue; nanocellulose in the lumen of the defect with signs of myxoid degeneration (asterisk) with active osteonductive potential, and with the formation of a diffuse shaft of bone tissue along the periphery of the composite (arrows). (a–c) Hematoxylin and eosin staining, 100× magnification, scale bar 500 μm. (d–f) Histochemical staining of collagen proteins with Masson's trichrome, 100× magnification, scale bar 500 μm. NFC, nanofibrillated cellulose‐based composite with dicalcium phosphate dihydrate.

**On Day 30** (Table 1, Figure 3),

In the ABC group, a progressive increase in formed bone tissue with randomly located Haversian canals and wide bone trabeculae with numerous extensive bridge‐like contacts was observed, accounting for 91.9% of the length and 71.8% of the thickness of the cortical plate. Cartilaginous tissue accounted for 8.1% and fibrous tissue amounted to 10.3%.

In the NFC‐1 group, the closure of the defect with newly formed bone tissue was 21.9% of the length and 31% of the thickness of the cortical plate; the composite accounted for 39.9% of the cortical plate defect, cartilaginous tissue amounted to 13.5% and fibrous tissue composed 9.8% of the cortical plate defect.

In the NFC‐2 group, the closure of the defect with newly formed bone tissue was more than 92% of the length and thickness of the cortical plate. In total, 3.6% of other tissues were cartilaginous tissue and 1.6% were fibrous tissue.

**Figure 4:**
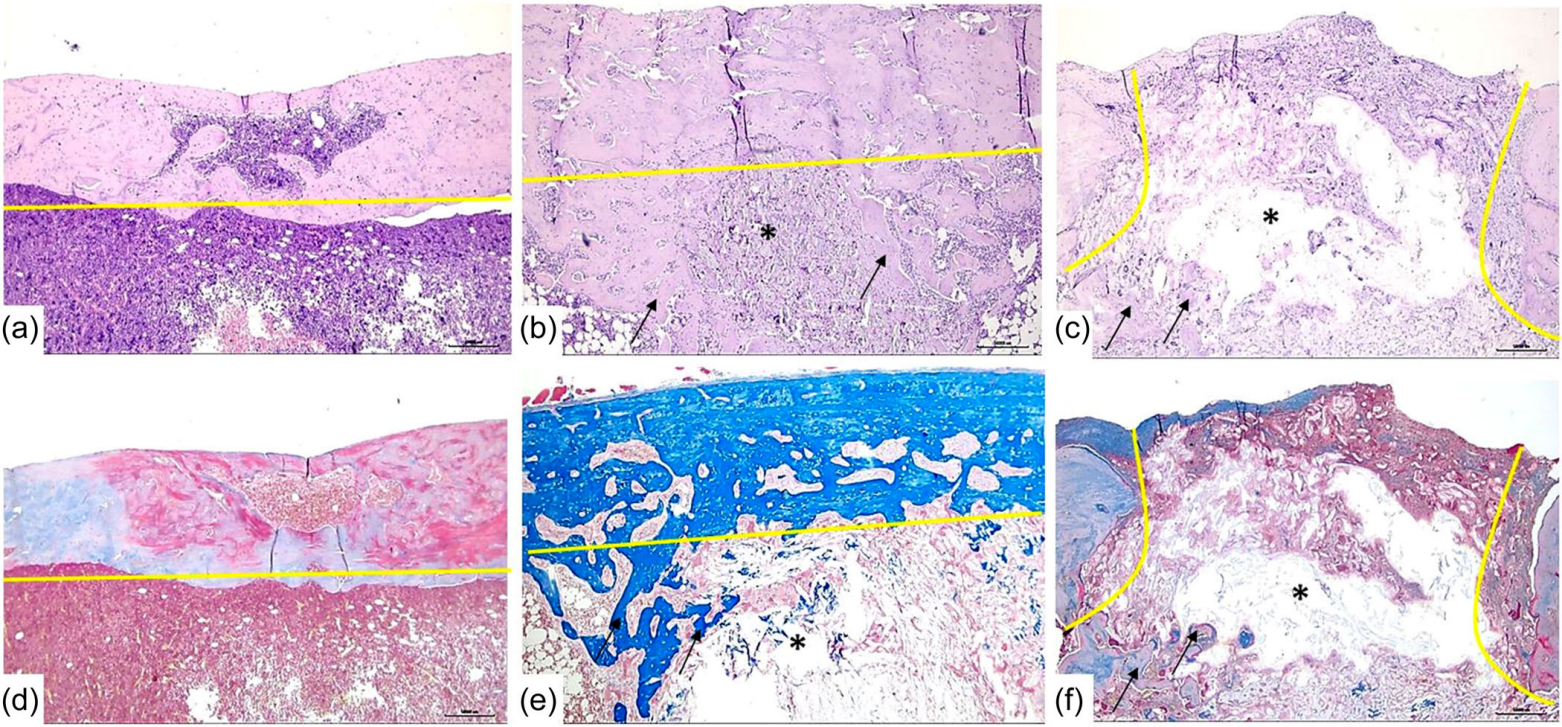
Representative photomicrographs of the defect area of the cortical plate and intramedullary space on the 60th day after filling the defect with an autologous blood clot (ABC) and nanocellulose with different filling techniques. (a, d) Photomicrographs of the defect area with ABC filling—The defect area is completely closed by newly formed bone tissue, with complete regeneration of the continuity of the cortical plate length and thickness in the defect area (in yellow is a straight line showing the uniform regeneration of the cortical plate thickness in relation to the edges of the defect). Mature bone marrow in the intramedullary space indicates a positive remodelling of the intramedullary space in the defect area with the regeneration of the normal anatomical structure. (b, с, е, f) Photomicrographs of the defect area filled with nanocellulose in various modifications: NFC‐1 and NFC‐2. (b, e) NFC‐2 group: Filling of the intramedullary space defect with nanocellulose without filling the cortical plate defect; relatively uniform regeneration of up to 90% of the cortical plate defect area in length and thickness with newly formed bone tissue (a straight yellow line shows uniform and complete integration of the defect edges with the newly formed bone tissue); in the intramedullary space, under the cortical plate, a nanocellulose‐based composite (asterisk) with a large number of bone trabeculae (arrows) along the periphery and in the centre of the composite is observed, which reflects active osteogenesis. (c, f) NFC‐1 group: Filling the defect with nanocellulose both in the intramedullary space and in the cortical plate; relatively uneven and partial regeneration of the cortical plate defect area with newly formed bone tissue without histo signs of a positive reparative process compared to 30 days (Figure 3) (a discontinuous yellow line shows incomplete integration of the defect edges with the newly formed bone tissue; nanocellulose obturating the defect lumen with signs of myxoid degeneration (asterisk) with active osteoinductive potential, and with the formation of bone along the periphery of the composite, chaotically growing into the intramedullary space (arrows). (a–c) Hematoxylin and eosin staining, 100× magnification, scale bar 500 μm. (d–f) Histochemical staining of collagen proteins with Masson's trichrome, 100× magnification, scale bar 500 μm. NFC, nanofibrillated cellulose‐based composite with dicalcium phosphate dihydrate.

**On Day 60** (Table 1, Figure 4),

In the ABC group, the bone tissue consisted of orderly located bone trabeculae that merged into lamellae. The closure of the defect with newly formed bone tissue was more than 90% of the length and thickness of the cortical plate. Cartilaginous tissue accounted for 4%, and fibrous tissue amounted to 2.9%.

In the NFC‐1 group, the closure of the defect with newly formed bone tissue was 26.9% of the length and 44.4% of the thickness of the cortical plate; the composite accounted for 42.3% of the cortical plate defect, cartilaginous tissue amounted to 18.9%, and fibroАрus tissue composed 20.3% of the cortical plate defect.

In the NFC‐2 group, the closure of the defect with newly formed bone tissue was more than 92% of the length and thickness of the cortical plate. In this case, the defect area is represented by dense mineralized bone tissue containing Haversian canals of different sizes. Less than 10% of other tissues consisted of cartilaginous tissue, accounting for 3.6%, while 1.6% consisted of fibrous tissue.

#### Comparative assessment of inflammatory and necrotic patterns

After 14 days, both in the ABC and NFC‐2 groups, a single reactive cellular infiltration without necrotic changes was observed in the defect area. After 30 and 60 days, no signs of inflammatory cell infiltration, infection indicators (polymorphonuclear cells, lymphocytes, macrophages or multinucleated cells) or tissue necrosis were observed in any of the surgically created defects.

### Intramedullary space

**On Day 14** (Table 1),

In the ABC group, more than 75% of the intramedullary space was occupied by bone marrow, approximately 10% by bone and cartilaginous tissue, and around 5% by fibrous tissue.

In the NFC‐1 group, one third (30.8%) of the intramedullary space was occupied by the composite, one fifth (18.3%) by bone tissue, and approximately 10% by cartilaginous and fibrous tissue, as well as bone marrow.

In the NFC‐2 group, one fifth (20.5%) was occupied by the composite, approximately one third by bone tissue (29.1%) and bone marrow (33.9%), and less than 10% by cartilaginous and fibrous tissue.

**On Day 30** (Table 1),

In the ABC group, more than 90% of the intramedullary space was occupied by bone marrow, and approximately 10% by other tissues, of which 5% was bone tissue, 2.6% was cartilaginous tissue and 1.4% was fibrous tissue.

In the NFC‐1 group, one third of the intramedullary space (29.5%) was occupied by composite and bone tissue (29.7%), while approximately 20% by cartilaginous and fibrous tissue.

In the NFC‐2 group, one fifth (19%) of the intramedullary space was occupied by the composite, 42.3% were composed of bone tissue and bone marrow (21.4%), and less than 10% were composed of cartilaginous and fibrous tissue.

**On Day 60** (Table 1),

In the ABC group, more than 90% of the intramedullary space was occupied by the bone marrow and approximately 10% by bone, cartilaginous tissue and fibrous tissue.

In the NFC‐1 group, one third of the intramedullary space (32.3%) was occupied by composite and bone tissue (32%), and other tissues made up approximately one third, of which cartilaginous tissue accounted for 13.3%, fibrous tissue amounted to 14.6% and bone marrow composed 7.9% of the intramedullary space.

In the NFC‐2 group, one fifth (19.1%) of the intramedullary space was occupied by the composite, approximately one third was made up of bone tissue (37.6%), one quarter was composed of bone marrow (25.1%) and less than 10% were cartilaginous and fibrous tissues (8.4% and 9.8%, respectively).

## DISCUSSION

In this study, the healing of a femoral bone defect was assessed using a nanofibrillated cellulose‐based composite with dicalcium phosphate dihydrate, in comparison with an ABC. The assessment included histopathological and histomorphometric analyses.

Our results showed that the use of the NFC‐1 defect‐filling technique significantly decreases the rate of osteogenesis in the cortical plate compared with that in the ABC and NFC‐2 groups (*p* < 0.05). In particular, in the NFC‐1 group, the closure of the cortical plate defect was mainly represented by the vertical growth and maturation of bone tissue along the edge of the cortical plate defect. Horizontal bone growth was characterized by insufficient formation of newly formed bone tissue with closure of defect area by less than 30% on Day 30. Thus, on the 14th, 30th and 60th days, a significantly smaller amount of newly formed bone tissue within the cortical plate was observed, compared to the ABC and NFC‐2 groups (*p* < 0.01).

In the NFC‐1 group, in the defect area, an unremodeled composite, surrounded by fibrous tissue with a weak cellular infiltrate, weakly integrated with the newly formed bone tissue, was observed. The absence of bone and cartilaginous tissue, as well as blood vessels inside the composites, was also noted at the level of the cortical plate. Our results complement the existing data demonstrating the weak osteoconductive properties of nanocellulose [27, 47, 48].

Previously, we showed that the formation of a favourable reparative microenvironment with reactive lymphohistiocytic infiltration and well‐vascularized maturing granulation tissue is an important condition for the formation and homoeostasis of the bone lacunar‐canalicular system, and active integration of newly formed bone tissue with a composite [35, 36, 37].

The bone lacunar‐canalicular system plays a very important role in the regeneration of compact bone tissue: by connecting osteoblasts, osteocytes and blood vessels, it provides nutrition to bone cells and mineralization of bone tissue. We believe that the low osteoconductive effect of nanocellulose composites may be associated with early and faster biodegradation of the composite in the form of myxoid degeneration with the formation of amorphous glassy gel‐like mass, limiting the integration of the composite with the resulting bone tissue and the formation of the bone lacunar‐canalicular system.

An important result of the study is the detection of prolonged osteogenesis in the intramedullary space on Days 30 and 60 in NFC‐1 and NFC‐2 groups. In particular, in both groups, vertical growth of bone tissue was observed along the periphery and the edge of the nanocellulose composite in the intramedullary space. At the same time, both in the NFC‐1 and NFC‐2 groups, a significantly larger amount of bone tissue was observed in the intramedullary space on Days 14, 30 and 60 in comparison with the ABC group (*p* < 0.0001).

In the NFC‐2 group, on Day 30, a larger amount of bone tissue in the intramedullary space compared to the NFC‐1 group was observed (*p* < 0.0001).

Our results complement the existing data demonstrating that nanocellulose has osteoinductive potential [14, 23, 32]. It has been previously shown that nanocellulose supports the growth and differentiation of stem cells into osteogenic cell lines, and both unmodified and modified nanocellulose scaffolds support the adhesion and viability of mesenchymal stem cells [40].

Nishiguchi and Taguchi [28] evaluated the immune response generated by the nanocellulose hydrogel vaccine scaffold in mice. They implanted the antigen‐loaded hydrogels subcutaneously in mice and analysed the local tissue response. Histological observations showed that the nanofibrous scaffold recruited and infiltrated macrophages and dendritic cells into the implantation site. This result suggests the nanocellulose immune scaffold is able to enhance antigen‐specific cellular immune responses in mice.

We believe that in our study, the different osteoinductive potentials of the composites in the NFC‐1 and NFC‐2 groups were due to the size of the composites used intraoperatively. One disadvantage of utilizing nanocellulose‐based composites in the human organism is their low degradation rate due to the absence of the cellulase enzyme [21], coupled with poor mechanical properties. Earlier, cellulose degradation in rats was studied by Mattson et al. [24], who showed that cellulose‐based scaffolds, implanted in the subcutaneous tissue, were characterized by very slow degradation and remodelling. We believe that in some cases, the slow degradation of nanocellulose may be beneficial since it can have a long‐term/prolonged effect on the immune response, which is required for the regeneration of large bone defects. It can be assumed that the long‐term preservation of nanocellulose in the dynamic environment of the body provides prolonged osteoinductive potential, contributing to continued osteogenesis. In future research, the priority will be to understand the rate of degradation of these scaffolds in vivo and the corresponding cellular activity that leads to degradation and remodelling in the implant space.

The regeneration of bone defects is sometimes accompanied by bone necrosis zones [11]. Our results showed that at all stages of the study, there was no pronounced necrosis in both the NFS‐1 and NFC‐2 groups and in the ABC group. At the same time, a dense collagen capsule formed around the implants in the NFC‐2 group, which indicates the maturation of the tissue reaction. On Days 30 and 60, in the NFC‐2 group, histopathological analysis revealed a predominant pattern of complete repair of the cortical plate and intramedullary space in the defect area by mature bone tissue. In general, histological evaluation demonstrated a normal response to rat cellulose implants with an increase in fibrous encapsulation over time. Minimal inflammation and tissue integration with vascularization confirmed the high biocompatibility of the NFC‐based composite with the NFC‐2 filling technique for bone regeneration. This has promising implications not only for bone grafting but also for other orthopaedic scenarios requiring bone remodelling with minimal risk of necrosis.

The results of our study showed that in more than one third (38%) of cases in the NFC‐1 group, cartilaginous and connective tissue prevailed in the area of the cortical plate defect, and bone tissue was present in a minimal amount. We believe that the persistence of this pattern may be associated with the contact of the periosteum with the composite material and the intramedullary space with the formation of a fibroplastic barrier for osteogenesis, which reduces the reparative ability of the bone.

Our study also showed that, according to the histological data, there was no significant difference in the healing of the cortical plate between the ABC group and NFC‐2 group on Day 60. We assume that this result may be due to the completion of the active phase of osteogenesis, leading to full closure of the cortical plate in both the ABC group and NFC‐2 group on Day 60. However, bone tissue reconstruction and remodelling are complex processes that continue beyond the active phase of osteogenesis [25, 45]. These processes include the reorganization and rebuilding of newly formed bone tissue, ensuring its strength and functionality. Although complete closure of the bone defect can be achieved, ongoing reconstruction and remodelling may still occur [9, 18]. This ongoing process may contribute to the absence of a significant difference in newly formed bone tissue in the cortical plate between the ABC group and NFC‐2 group, observed on Day 60. This suggests the need for additional research to fully understand these processes. In addition, we showed that in the NFC‐2 group and ABC group, there were no bone repair disorders associated with insufficient bone formation within the intramedullary space and cortical plate. The newly formed bone tissue in both groups was characterized by normal histoarchitecture: the cortical plate had a laminar‐layered structure with a focal chaotic pattern in the area of predominantly Haversian canals. The relative amount of bone tissue and Haversian canals did not differ both between the groups and from the bone structure outside the bone defect area.

On the contrary, the NFC‐1 group demonstrated worse cortical plate healing rates compared to the ABC group. The results of our study showed that in more than one third (38%) of cases in the NFC‐1 group, cartilage and connective tissue prevailed in the defect area, and bone tissue was present in minimal amounts. We believe that the preservation of this pattern may be due to the contact of the periosteum with the composite material and the intramedullary space with the formation of a fibroplastic barrier for osteogenesis, which reduces the reparative ability of the bone. In addition, lower rates in the NFC‐1 group may be associated with insufficient vascularization and altered mechanical pressure in the cortical region, which further limits effective bone regeneration.

The key advantage of this study is the comparative assessment of the use of the NFC‐based composite transplanted into a bone defect in comparison with an ABC both at the early (Day 14) and late stage (Day 60) of bone defect reparation. This approach contributed to the discovery of prolonged osteoregeneration on Day 60 in the intramedullary space using the NFC‐based composite. The use of a standardized model of a rat femoral defect, along with histological and morphometric analysis, further increases the reliability of the study.

### Limitations

The evaluation of bone regeneration was based solely on histological methods, which, although informative, do not completely exhaust all potential analytical methods. These limitations should be taken into account when interpreting the results and influence the design of future research aimed at filling potential knowledge gaps.

## CONCLUSIONS

Thus, our experimental study showed that, depending on the intraoperative method of nanocellulose‐based composites implantation into the bone defect area, it can both improve and/or worsen the reparation of the bone tissue defect. We found that on the 30th and 60th days, the NFC‐2 group (with filling of only the intramedullary space in the defect area) showed a predominant pattern of complete repair of the cortical plate and continued bone growth in the intramedullary zone characterized by a large volume of newly formed bone in the intramedullary space and a circular pattern of newly formed bone. We believe that the main effect of prolonged osteoinductive potential is associated with very slow degradation and decomposition of the NFC‐based composite, which promotes continued growth and maturation of bone tissue in the intramedullary space.

## AUTHOR CONTRIBUTIONS

The applicant's contribution was direct participation in experimental work, development of operational techniques for nanomaterial transplantation, preparation of materials, breeding of experimental animals and processing of experimental research data.

## CONFLICT OF INTEREST STATEMENT

The authors declare no conflict of interest.

## ETHICS STATEMENT

The study was conducted according to the European Convention for the Protection of Vertebrate Animals Used for Experimental and Other Scientific Purposes (2010) and approved by the Local Ethics Committee of Karaganda Medical University (No. 18 20.09.2022).

## Data Availability

The data sets generated and/or analyzed during the current study are available from the corresponding author upon reasonable request.
